# Nutritional evaluation of stage 5 chronic kidney disease patients on dialysis

**DOI:** 10.1590/S1516-31802012000600006

**Published:** 2013-01-18

**Authors:** Cynthia Mauro Piratelli, Rodolpho Telarolli

**Affiliations:** I MD. Director of the Medical College, Centro Universitário de Araraquara (Uniara), Araraquara, São Paulo, Brazil.; II MD, PhD. Adjunct Professor in the Department of Biological Sciences, Universidade Estadual Paulista (Unesp), Araraquara, São Paulo, Brazil.

**Keywords:** Renal insufficiency, chronic, Renal dialysis, Protein-energy malnutrition, Nutrition assessment, Nutrition disorders, Insuficiência renal crônica, Diálise renal, Desnutrição proteico-calórica, Avaliação nutricional, Transtornos nutricionais

## Abstract

**CONTEXT AND OBJECTIVE:**

Patients with chronic kidney failure undergoing dialysis have high prevalence of protein-energy malnutrition. There is still no uniform method for assessing these patients’ nutritional status. It is recommended that a set of subjective and objective methods should be applied so that an adequate nutritional diagnosis can be reached. The aim of this study was to evaluate the nutritional profile of patients undergoing hemodialysis.

**DESIGN AND SETTING:**

Cross-sectional study conducted in the Dialysis Treatment Unit, Araraquara, São Paulo, Brazil, in 2008.

**METHODS:**

Anthropometric and biochemical indicators were characterized for 48 patients who also gave responses to the modified Subjective Global Assessment questionnaire (SGAm), and possible correlations between these indicators were investigated.

**RESULTS:**

The frequency of moderate or severe malnutrition ranged from 22% to 54%, according to the parameter used. Regarding the patients’ conformity with the ideal weight, 29% of them weighed less than 75% of the ideal, and thus were classified as having moderate or severe malnutrition. The most significant correlations were observed between body mass index (BMI) and the idealness of triceps skinfold (TSF), upper arm circumference (UAC) and upper arm muscle circumference (UAMC); and between SGAm and the idealness of UAC and UAMC.

**CONCLUSION:**

The frequency of malnutrition showed great variability among the patients, according to the evaluation criterion chosen. Routine nutritional monitoring and validation of methods for assessing body composition among such patients are extremely important for diagnosing malnutrition early on, thus preventing complications and reducing the morbidity and mortality rates in this population.

## RESUMO

**CONTEXTO E OBJETIVO:**

Portadores de insuficiência renal crônica em diálise apresentam alta prevalência de desnutrição proteico-energética. Não existe ainda um método uniforme para avaliar o estado nutricional desses pacientes. Recomenda-se a aplicação de um conjunto de métodos subjetivos e objetivos para se chegar aos diagnósticos nutricionais adequados. O objetivo deste estudo é traçar o perfil nutricional de pacientes submetidos a hemodiálise.

**TIPO DE ESTUDO E LOCAL:**

Estudo transversal descritivo realizado na Unidade de Tratamento Dialítico de Araraquara, São Paulo, Brasil, em 2008.

**MÉTODOS:**

48 pacientes tiveram seus indicadores antropométricos e bioquímicos caracterizados, sendo também submetidos ao questionário Avaliação Global Subjetiva modificada (SGAm), verificando-se possíveis correlações entre esses indicadores.

**RESULTADOS:**

A frequência de desnutrição moderada e grave variou de 22% a 54%, de acordo com o parâmetro utilizado. Com relação à adequação do peso ideal, 29% da amostra estavam com porcentagem de adequação abaixo do percentil 75, classificados como portadores de desnutrição moderada e grave. As correlações mais significativas foram observadas entre índice de massa corporal (IMC) e adequações de prega triciptal (PCT), circunferência do braço (CB) e circunferência muscular do braço (CMB); e entre o SGAm e adequações de CB e CMB.

**CONCLUSÃO:**

A desnutrição apresentou grande variabilidade de frequência entre os pacientes de acordo com o critério escolhido para avaliação. O acompanhamento nutricional de rotina e a validação de métodos que avaliem a composição corporal desses pacientes são de extrema importância para diagnosticar precocemente a desnutrição e assim prevenir complicações e reduzir as taxas de morbimortalidade nesta população.

## INTRODUCTION

Patients with chronic kidney failure, which is often an indication for kidney replacement therapy, have high prevalence of protein-energy malnutrition. This is characterized by changes to serum proteins and imbalance between the protein and fat components of the organism.^1^^,^^2^ Among the main causes of protein-energy malnutrition in this population are changes to energy metabolism and calorie levels, hormonal disorders, poor food intake, anorexia, nausea and vomiting, relating to the constant state of inflammation and uremic toxicity and occurrences of concomitant infection and inflammation.^3^

Since good nutritional status among patients with chronic kidney failure is associated with reduction of comorbidities, protein-energy malnutrition is considered to be a marker of poor prognosis. The prevalence of malnutrition among chronic hemodialysis patients ranges from 10 to 70%,^4^ and this malnutrition has frequently been documented in terms of reduction of subcutaneous fat deposits and loss of muscle mass, as assessed using simple anthropometric methods, and in terms of reductions in the levels of body nitrogen, serum albumin, transferrin and other visceral proteins. Nutritional markers such as serum albumin and low body mass index are associated with higher mortality rates in this population.^5^

The ultimate goal is to ensure that adequate body fat levels are maintained. This is very important in relation to patients on hemodialysis, because at times of greater energy demand to which they are exposed (vascular access surgery, infections or even kidney transplant), the fat reserves can be used to supply the energy deficit, thereby safeguarding the patient’s protein reserves.^6^

The nutritional status of hemodialysis patients is still often neglected in many dialysis centers around the world, although the benefits of ascertaining the anthropometric and dietary history of this population are known.^7^ There is still no uniform method for evaluating these patients’ nutritional status, and what is recommended is that a set of subjective and objective methods (overall history, food intake, physical examination, anthropometric measurements and biochemical tests) should be applied in order to arrive at an adequate nutritional diagnosis.^8^

## OBJECTIVE

The aim of this study was to quantify the prevalence of malnutrition among patients with chronic kidney failure on hemodialysis in the city of Araraquara, São Paulo, Brazil, through subjective and objective criteria, including anthropometric, biochemical and subjective overall assessment.

## METHODS

This was a cross-sectional descriptive study conducted in a private dialysis unit in the municipality of Araraquara, São Paulo, Brazil. All patients who started dialysis treatment in June 2008 were eligible for the study. A total of 48 adult patients aged 18 to 80 years of both genders, with chronic kidney failure, underwent regular hemodialysis sessions for at least three months, three times a week. These patients received oral diet, did not present consumptive disease and had not undergone blood transfusion or iron therapy over the last thirty days before the survey.

Patients excluded from the study had a history of hospitalization over the last 30 days preceding the survey and/or were receiving parenteral nutrition support at the time of the study, or had a history of infection or sepsis during the study period, or were suffering from advanced senility or dementia that might interfere with the questionnaire.

The survey was conducted with the universe of patients seen in the dialysis unit, respecting the exclusion conditions. No kind of sampling was used. The patients underwent anthropometric nutritional assessment consisting of body weight measurements post-dialysis and measurements of height, upper arm circumference (UAC), upper arm muscle circumference (UAMC) and triceps skinfold thickness (TSF). They also provided responses to the modified Subjective Global Assessment (SGAm) questionnaire.

The post-dialysis weight was measured in kilograms at the end of the hemodialysis session, on the same day that the skinfolds were measured. The patients were assessed without shoes on and with as little clothing as possible. We used an electronic scale for the weight measurements (Filizola, São Paulo, Brazil). The patients’ heights were obtained using a stadiometer.

These heights were used in combination with the weights to calculate the body mass index (BMI) for the nutritional assessment. The BMI was calculated as the ratio of body weight to the height squared (kg/m^2^), and the patients’ nutritional status was classified as follows, according to the BMI:^4^ adequate (24.1 to 30), mild malnutrition (22-24), moderate malnutrition (19 to 21.9) or severe malnutrition (< 19). The percentage adequacy of nutritional status was calculated as the ratio between dry weight and measured weight (x 100). The ideal weight was calculated by multiplying the desired BMI (we used the value of 24.1 for all patients) by the height squared. The patients’ nutritional status was then classified as described by Nelson et al.^9^

The TSF was measured with the aid of an adipometer, two inches above the midpoint between the acromial process of the scapula and the olecranon. The UAC was measured (in centimeters) using an inelastic and non-extendable tape of length 150 cm, graduated in divisions of 0.1 cm, at the midpoint of the extended upper arm, i.e. at the same site where the TSF was obtained. All measurements were made on the opposite side of the arm to the arteriovenous fistula. The UAMC was calculated using the formula: UAMC (cm) = UAC (cm)-3.14 x TSF (cm).

The UAC, UAMC and TSF data were then compared with the data from the National Health and Nutrition Examination Survey (NHANES I), as shown in tables of percentiles by Frisancho,^10^ including for elderly patients. After adjustments were made to the 50^th^ percentile, nutritional status was classified in accordance with Blackburn and Thornton.^11^

The biochemical evaluation included recording pre-dialysis albumin and transferrin, in order to classify the patients using the Riella-Martins standards.^1^ To evaluate nutritional supplementation, the Subjective Global Assessment, as modified by Kalantar-Zadeh et al., was applied.^12^

The patients’ nutritional characteristics were analyzed using descriptive statistics, with tabulation using the Microsoft Excel software. Frequency distributions of absolute and relative variables of interest were then analyzed according to gender. The data were also analyzed to make correlations between quantitative variables, by calculating the correlation coefficient (r).

## RESULTS

The study population consisted of 31 male (64.60%) and 17 female patients (35.40%), with ages ranging from 23 to 75 years. According to their BMI, 54.16% of the patients were classified as presenting moderate or severe malnutrition (BMI < 24.1),^4^ and among these, 57.69% were women and 42.30% were men. Also in relation to BMI, 35.42% of the patients had values between 24.1 and 30.0.

Regarding the patients’ conformity with the ideal weight, 29.16% of them weighed less than 75% of the ideal, and thus were classified as having moderate or severe malnutrition. Among these, 64.28% were men and 35.71% were women. At the other extreme, we found that 27.08% were obese, weighing more than 115% of the ideal.

Using the criterion of idealness of triceps skinfold thickness, 52.09% of the patients were classified as obese, of whom 80.65% were men. On the other hand, among the women, it was observed that 70.58% had some degree of malnutrition according to the same criterion, with 23.53% showing mild malnutrition, 11.76% moderate and 35.29% severe. Only 9.68% of the men evaluated had malnutrition according to this criterion, i.e. a ratio of four men for each woman. It should be noted that according to this criterion, the frequency of obesity was much greater than the frequency of malnutrition **(**Table 1**)**.

**Table 1.** Distribution of hemodialysis patients according to the idealness of triceps skinfold thickness (TSF), upper arm circumference (UAC) and upper arm muscle circumference (UAMC), used as criteria for classifying nutritional status

Variable	Severe malnutrition		Moderate malnutrition		Mild malnutrition		Eutrophic status		Overweight		Obesity	
	n	%	n	%	n	%	n	%	n	%	n	%
TSF												
Total	9	18.75	2	4.17	4	8.33	7	14.58	1	2.08	25	52.09
Female	6	35.29	2	11.76	4	23.53	5	29.41	-	0.00	-	0.00
Male	3	9.68	-	0.00	-	0.00	2	6.45	1	3.22	25	80.65
UAC												
Total	3	6.25	11	22.92	11	22.92	20	41.67	2	4.17	1	2.07
Female	1	5.88	5	29.41	2	11.76	8	47.06	1	5.88	-	0.00
Male	2	6.45	6	19.35	9	29.03	12	38.71	1	3.23	1	3.23
UAMC												
Total	5	10.42	15	31.25	11	22.92	13	27.08	4	8.33	-	0.00
Female	-	0.00	3	17.65	4	23.53	7	41.18	3	17.65	-	0.00
Male	5	16.13	12	38.71	7	22.58	6	19.35	1	3.23	-	0.00

According to the criterion of idealness of upper arm circumference, 52.09% were classified as malnourished, and obesity was present in only one patient in this sample (2.07%). Among the men, 54.83% were classified as malnourished, while 47.0% of the women were thus classified **(**Table 1**)**. In total, 41.67% of the men and women presented upper arm circumference within the range of 90-110% of the ideal. According to this criterion, malnutrition was eight times more frequent than overweight and obesity.

According to the criterion of idealness of upper arm muscle circumference, 64.5% had some degree of malnutrition, among which 16.13% had mild malnutrition, 35.49% moderate and 48.38% severe. Among the women studied, 41.18% were adequate according to this criterion and another 41.18% were malnourished. Among the men, the percentage of malnutrition was 77.41% **(**Table 1**)**.

According to the laboratory criteria of protein reserves, the results showed that 58.34% of the patients had mild malnutrition, consisting of plasma albumin between 3.0 and 3.9 g/dl. Among these, 64.28% were male and 35.72% were female. The plasma transferrin levels classified the vast majority of the patients (89.58%) as having adequate nutritional status. Among all the women, only one case presented a state of severe malnutrition (transferrin less than 100 mg/dl), and among the 4 malnutrition cases in men according to this criterion, 75% had mild to moderate malnutrition and 25% had severe malnutrition **(**Table 2**)**.

**Table 2.** Classification of nutritional status among hemodialysis patients, according to the criteria of plasma albumin and transferrin

Variable	Severe malnutrition		Moderate malnutrition		Mild malnutrition		Eutrophic		Total
	n	%	n	%	n	%	n	%	
Albumin									
Total	-	0	1	2.08	28	58.34	19	39.6	48
Female	-	0	1	5.88	10	58.82	6	35.3	17
Male	-	0	-	0	18	58.06	13	42.0	31
Transferrin									
Total	1	2.08	1	2.08	3	6.25	43	89.58	48
Female	1	5.88	-	0	-	0	16	94.12	17
Male	-	0	1	3.23	3	9.68	27	87.1	31

Using the modified Subjective Global Assessment (SGAm) as a criterion for classifying nutritional status, it was seen that almost 100% of the patients were at nutritional risk of mild malnutrition. A single female patient had a score greater than 23, and was classified as moderately malnourished.

## DISCUSSION

Establishing patients’ nutritional status is currently an extensive and complex process despite the simplicity of the techniques and the low cost. The evaluation methods are not very accurate or very sensitive to environmental changes. In patients with chronic kidney disease, such evaluations also present greater difficulty because this kind of patient is much more susceptible to these and other variations such as metabolic and hydro-electrolytic factors, and in relation to the adequacy of their dialytic type.^13^

In the present study, the frequency of moderate or severe malnutrition ranged from 22% to 54%, depending on the criterion used. It was 54.16% according to BMI (kg/m^2^) and 29.16% according to the criterion of percentage of the ideal weight.

With regard to the idealness of other parameters, we found that 22.91% of the patients presented TSF within the ideal range, and 29.16% to 41.66% for UAC and UAMC. The biochemical parameter of plasma albumin showed that 58.33% of the sample were classified as presenting mild malnutrition, while transferrin showed that 89.58% of the sample was normal. The modified SGAM questionnaire showed that almost 100% of the sample presented mild or moderate malnutrition.

For populations of chronic kidney disease patients, the eutrophic BMI range (24.1 to 30) includes levels at which the general population would be classified as healthy overweight (25 to 29.9). This can be explained by the evidence of fluid retention in the kidney disease population and by the fact that hemodialysis patients with higher BMI have better survival,^4^ thus reinforcing the idea that it is preferable for BMI to be kept higher among such patients. In the present study, BMI showed a strong positive correlation with the idealness of UAC (r = 0.894) and with measurements of UAC (r = 0.75), and a significant correlation with the idealness of UAMC and TSF (r = 0.57 for both).

From assessment of the idealness of triceps skinfold thickness, the frequency of obesity in the sample was shown to be higher (52.08%) and these individuals were all male (n = 25), and most of them were over 45 years of age. From the same criterion among the women, 70.5% were classified as undernourished, and most of these were also over 45 years of age. According to this criterion, obesity was prevalent among men and malnutrition among women. This is in agreement with the literature, in which an excess of body fat among male kidney disease patients has been demonstrated.^14^ On the other hand, a study conducted in the state of Amazonas^15^ on 165 hemodialysis patients found reversed proportions, such that 38% of the women and 27% of the men presented TSF values below the 5^th^ percentile. This result was attributed to inadequate protein and calorie intake in that region of Brazil.

In a nutritional analysis on 16 patients with chronic kidney disease undergoing peritoneal dialysis, Koehnlein et al.^14^ showed that excess fat mass was observed more frequently among men. In our study, the results from UAC measurements, which usually reflect the organism’s protein and fat reserve composition, showed that 41.67% of the patients presented measurements that were close to the ideal, while 52.09% showed differing degrees of malnutrition, 6.24% showed excess weight and 6.25% showed obesity.

The idealness of AC correlated positively with the idealness of TSF (r = 0.44). TSF showed that 64.58% of the patients were malnourished and, among these, 77.41% were males, thus showing that muscle mass deficits were more common among men. This pattern was also found in the study by Valenzuela et al.,^15^ in which the degree of idealness of TSF was significantly lower among men than among women. This was also seen in the study by Koehnlein et al.,^14^ among patients on continuous ambulatory peritoneal dialysis, such that only the men in the sample had mild or moderate malnutrition in relation to TSF. Valenzuela et al.^15^ found that 39% of the men and 2% of the women presented with CMB values below the percentile 5, showing also a loss of muscle mass in man. In a study on nutritional assessment of renal patients on hemodialysis in São Luiz do Maranhão, UAC, UAMC and TSF showed that malnutrition was present in over 60% of the patients.^16^ The ferritin and hemoglobin levels proved to be adequate in 54.17% and 52.08% of the patients, respectively. These numbers were due to constant laboratory monitoring of these patients, with regard to these indicators. These results are consistent with a Portuguese^17^ study involving 75 patients in chronic hemodialysis program, among whom transferrin stocks proved to be adequate in 89.58% of the population.

Regarding the classification of the sample using the modified SGAm criteria, practically the entire patient sample was seen to be at risk of mild or moderate malnutrition. There was a weak negative correlation with the absolute TSF, UAC and UAMC values as well as with their adequacy criteria. Moreover, there was no correlation with other anthropometric and biochemical parameters. SGAm proved to be a simple and sufficiently sensitive instrument for identifying nutritional risk in this population, but it was also prone to false-negative results due to the dependent items of patients’ histories, a situation similar to what was reported by Vegine et al.^18^ The item on functional disability associated with nutritional status was the one that contributed least to the final score of the questionnaire, with an impact of 8.61%, while dialysis was the largest contributor, with 21% impact. Another noteworthy point in relation to the SGAm was its lack of sensitivity for detecting greater severity of malnutrition, as shown by the BMI criteria (54.16% with moderate and severe malnutrition) and UAC criteria (41.66% with moderate and severe malnutrition).

The main limitation of this study relates to the fact that it was based on a single measurement of anthropometric and biochemical parameters, made before the start of the replacement renal therapy. For greater precision, anthropometric evaluations must be performed continuously among patients on hemodialysis, so that over a period of time, these patients can serve as their own control, given that there are no specific reference values for patients in this condition. The same applies to the biochemical parameters, which are more subject to errors when punctually documented, as performed in this research. Specifically for albumin, the consequence of its long half-life is a slow response to acute protein depletion, which therefore leads to underestimation of declines in its serum levels. Use of nutritional criteria in isolation for diagnosing the status of hemodialysis patients may lead to significant errors, due to the diversity of pathological conditions to which hemodialysis patients are exposed, especially with regard to water retention and secondary metabolic disorders. Hence, it is important to simultaneously use several clinical and laboratory methods for nutritional assessment. It is important for these results to be validated by other studies of this type, since the present data was based on the population at a single dialysis center, with a relatively small number of patients, using anthropometric assessments and measurement of biochemical parameters after starting the dialysis process.

## CONCLUSIONS

The frequency of malnutrition showed great variability among the patients, according to the diagnostic criteria used, and ranged from 22% to 54% of the population. This variation can be explained by the fact that anthropometric measurements based on a two-compartment model (muscle mass and body fat) assume that, as with body fat, all components of lean body mass (water, proteins and minerals) are in the same proportions for all patients. For patients with chronic kidney failure that is not true, as a result of hydro-electrolyte changes in their condition. According to the BMI, 54.16% of patients were considered to be malnourished. Laboratory parameters relating to protein reserves showed conflicting results, such that the parameter of plasma albumin showed that 58.34% were malnourished, while plasma transferrin showed that only 10.41% were in this condition. The modified Subjective Global Assessment showed that all the patients were at risk, such that 97.92% presented a mild risk of malnutrition.
